# Liquid Levothyroxine Formulation Taken during Lunch in Italy: A Case Report and Review of the Literature

**DOI:** 10.1155/2020/8858887

**Published:** 2020-09-05

**Authors:** Carlo Cappelli, Ilenia Pirola, Maurizio Castellano

**Affiliations:** Department of Clinical and Experimental Sciences, SSD Medicina Ad Indirizzo Endocrino-metabolico, University of Brescia, ASST Spedali Civili di Brescia, Brescia 25123, Italy

## Abstract

Levothyroxine (L-T4) is among the most widely prescribed medications in the world, and it is considered by the World Health Organization an essential medicine for basic health care. Replacement therapy has always been considered straightforward although different factors may interfere with intestinal absorption of L-T4, including food, dietary fibre, coffee, drugs, and gastrointestinal diseases. For these reasons, current guidelines recommend that L-T4 should be taken in a fasting state because its absorption is maximised when it is taken on an empty stomach, reflecting the importance of gastric acidity in the absorption process. In addition to sodium L-T4 in tablet form, various formulations (soft-gel capsules and liquid solutions) have become available for clinical use in the last years promising improved absorption. We described a 31-year-old Italian man who took liquid levothyroxine formulation during lunch. He was under replacement therapy with liquid levothyroxine 75 mcg daily for hypothyroidism due to Hashimoto thyroiditis for three years. During confirmation of the L-T4 replacement therapy, the patient stated that he was going to continue to “take liquid levothyroxine during (his) lunch every day.” We recommended taking the medication correctly in the morning at least thirty minutes before breakfast and repeating TSH, fT4, and fT3 after three months. The thyroid hormonal profiles taken after 3 and 6 months were comparable to those when the patient was taking the medication during lunch. In conclusion, liquid levothyroxine formulation should be preferred in case of malabsorption or potential malabsorption. Liquid formulation should be preferred due to the possibility of taking it during breakfast, which significantly improves the compliance of patients. Further studies are needed to evaluate the possibility of taking liquid L-T4 during lunch.

## 1. Introduction

Levothyroxine is among the most widely prescribed medications in the world, and it is considered by the World Health Organization an essential medicine for basic health care [1–3].

The first use of thyroid hormone for the treatment of hypothyroidism was documented in the 1890s, when Bettencourt and Serrano described a patient grafted with an ovine thyroid gland to treat severe hypothyroidism [4]. Synthetic formulations of thyroxine have been available for use since the 1950s although desiccated animal thyroid gland remained the mainstay of therapy until the 1970s [5].

In addition to sodium levothyroxine (L-T4) in tablet form, today various L-T4 formulations (soft-gel capsules and liquid solutions) are being used in most but not all countries for clinical use in the last years [6]. Approximately 60–90% of a tablet L-T4 dose is absorbed within 3 h of ingestion, and the absorption is maximal when it is taken on an empty stomach, reflecting the importance of gastric acidity in the process. Differently, many reports showed that liquid formulations circumvent gastric acidity, improving their absorption even if ingested with breakfast [7–11].

We described a young Italian man who took liquid levothyroxine formulation during lunch.

## 2. Case Presentation

**Figure 1:**
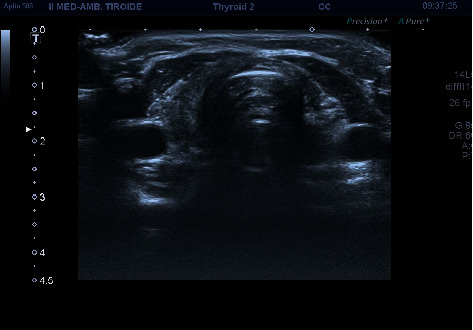
Thyroid ultrasound revealed an atrophic gland without nodules.

A 31-year-old male patient was referred to our Department for hypothyroidism on 3 April 2018. His medical history was not significant. He suffered from Hashimoto's thyroiditis and had been under replacement therapy with liquid levothyroxine 75 mcg daily (Tirosint 75 mcg-IBSA®) for three years. Physical examination showed no abnormalities. He exhibited two thyroid hormonal profiles (TSH, fT4, and fT3) taken, respectively, in the previous fifteen days and 6 months by outpatient evaluation (Table 1). The patient underwent thyroid ultrasound that showed an atrophic gland with marked hypoechoic tissue, without nodules (Figure 1). During confirmation of the L-T4 replacement therapy, the patient stated that he was going to continue to “take liquid levothyroxine during (his) lunch every day.” The patient said that, only during the first year, he had taken liquid L-T4 at least 30 minutes before breakfast, while in the last two, he had been taking liquid L-T4 with a glass of water during his lunch “for convenience.” We recommended taking the medication correctly in the morning at least thirty minutes before breakfast and repeating TSH, fT4, and fT3 after three months. The patient was reassessed on 27 July. He was in good health. The thyroid hormonal profiles are reported in Table 1. No difference in TSH, fT4, and fT3 was observed compared to the values found when the patient took the medication during lunch. New examinations were performed in the same laboratory after 6 months; TSH, fT4, and fT3 were still superimposable with the previous values (Table 1).

## 3. Discussion

We described for the first time, a patient taking liquid L-T4 during lunch in Italy. There was no change in serum TSH level after changing the timing of L-T4 ingestion to at least 30 minutes before breakfast.

Until ten years ago, the only formulation available was the tablet. Replacement therapy has always been considered straightforward although different factors may interfere with intestinal absorption of L-T4, including food, dietary fibre, coffee, drugs, and gastrointestinal diseases [12]. For these reasons, current guidelines recommend that L-T4 should be taken in a fasting state at least 30 minutes before breakfast or at bedtime (at least three hours after the evening meal) because its absorption is maximised when it is taken on an empty stomach, reflecting the importance of gastric acidity in the absorption process. [13, 14]. Indeed, discordant results on timing of L-T4 administration with respect to main meal are reported [15, 16].

Ten years ago, pharmaceutical companies introduced new levothyroxine formulations (liquid and soft-gel capsules) promising improved absorption. This could be due to the fact that these formulations circumvent the phase of gastric dissolution, which is closely dependent on gastric pH. This is true for liquid formulations because the active ingredient is already dissolved in 85% glycerol and 96% ethanol. Cassio et al. showed that, for the first time in 2013, there is no full bioequivalence between drops and tablets, especially for infants with severe congenital hypothyroidism, suggesting that liquid form could be more effective and/or better absorbed than tablets [17]. The following year, our group observed by chance a series of euthyroid patients who wrongly took liquid L-T4 with coffee at breakfast; after changing the time of intake to 30 min before breakfast, no change in thyroid hormonal profile was observed. This feedback suggested that that liquid T4 can be taken orally at breakfast both with water and with coffee. Taking into account these data, we hypothesized that high temperatures (i.e., coffee temperature) do not alter the molecular properties or stability of L-T4 [10]. This was later clearly demonstrated by Bernareggi and colleagues [18]. Moreover, the TICO study, a double-blind placebo-controlled crossover trial, confirmed that liquid L-T4 can be ingested directly at breakfast, thus potentially improving therapeutic compliance [9], data recently confirmed in more than seven hundred patients [10].

Many other subsequent studies and observations have shown the significant superiority in terms of TSH normalization of the liquid formulation compared to tablets even in different subsets of patients, such as those with or without gastrointestinal malabsorption, submitted to bariatric surgery or taking multiple concomitant drugs [19–24]. Furthermore, the use of liquid L-T4 showed a significantly reduced variability in TSH values, both in young and older people, with a higher number of patients who remained euthyroid during follow-up [25, 26]. This is relevant for at least two reasons: firstly, the simultaneous reduction in risk of developing other disorders mainly associated, but not exclusive, with clinical or subclinical hyperthyroidism, such as atrial fibrillation, osteoporosis, and coronary heart disease [27, 28]; secondly, subjects with stable euthyroidism during replacement L-T4 therapy required fewer blood checks, with a predictable relative reduction in total health care expenditure [29, 30].

Finally, the possibility of taking levothyroxine treatment during breakfast improves quality of life [31] and significantly improves adherence to the treatment [27] as recently reported by two Italian surveys.

Differently from liquid solution, soft-gel capsules contain the drug dissolved in glycerin and enclosed in a gelatinous matrix. This structure should promise protection from variations in gastric pH. In agreement, in vitro research showed that the dissolution profile was more consistent than for tablets in the entire pH range after 60–120 minutes [32]. This has been confirmed in vivo by Fiorni et al., showing a total dissolution time of about 20 minutes [33]. Literature shows that this formulation improves the TSH profile in patients with impaired gastric secretion [34], taking pump inhibitors [35], with central hypothyroidism [36], in postmenopausal woman taking calcium supplements [37] and also a few minutes before breakfast with coffee [38], as compared to tablets. On the contrary, Di Donna et al. demonstrated that there was no difference in L-T4 requirement between soft-gel capsules and tablets in patients without malabsorption although the serum TSH was lower in patients taking soft-gel capsules [39]. To the best of our knowledge only one study, the “TITI” study evaluated whether a soft-gel capsule of L-T4 could also be ingested at breakfast, instead of liquid formulation. The study showed that both the liquid and soft-gel capsule formulations of L-T4 can be taken with breakfast although a significant decrease in fT4 and fT3 was observed 6 months after the switch from liquid to soft-gel capsules [40]. For these reasons, the authors suggested that “liquid L-T4 would be the preferred formulation for patients in whom even small changes in fT4 and fT3 levels are to be avoided.” Further studies are needed to clarify this important issue.

## 4. Conclusion

The new levothyroxine formulation should be preferred in case of malabsorption or potential malabsorption. Liquid formulation should be preferred due to the possibility of taking it during breakfast, which significantly improves the compliance of patients. Further studies are needed to evaluate the possibility of taking liquid L-T4 during lunch.

## Figures and Tables

**Table 1:** Thyroid function tests while taking liquid levothyroxine during lunch and before breakfast at the same daily dose.

	Liquid L-T4 (75 mcg/die) taken during lunch		Liquid L-T4 (75 mcg/die) taken 30 minutes before breakfast	
	6 months before evaluation	15 days before evaluation	After 3 months from evaluation	After 9 months from evaluation
TSH, mIU/L (0.27–4.2)	1.71	1.69	1.80	1.75
fT4, pg/ml (8.0–19.0)	15.7	15.3	15.9	15.6
fT3, pg/ml (2.4–4.7)	3.3	3.1	3.4	3.3

## Data Availability

The data used to support the findings of the study are available on request.
